# Development and validation of a practical prediction model for post-ERCP pancreatitis using machine learning

**DOI:** 10.3389/fsurg.2025.1628956

**Published:** 2025-11-03

**Authors:** Tianyu De, Guohui Du, Hongkun Yin, Hao Wang, Wei Wang, Tian Ma, Junbai Ma, Hao Wang, Qi Wang

**Affiliations:** 1 Department of Hepatobiliary Surgery, General Hospital of Ningxia Medical University, Yinchuan, China; 2 School of Clinical Medicine, General Hospital of Ningxia Medical University, Yinchuan, China; 3 Neonatal Intensive Care, General Hospital of Ningxia Medical University, Yinchuan, China; 4 Department of Thoracic and Cardiovascular Surgery, General Hospital of Ningxia Medical University, Yinchuan, China; 5 School of Basic Medical Sciences, Ningxia Medical University, Yinchuan, China; 6 Department of Pathogenic Biology and Medical Immunology, School of Basic Medical Sciences, Ningxia Medical University, Yinchuan, China

**Keywords:** post-ERCP pancreatitis, artificial intelligence, predictive model, machine learning, risk prediction

## Abstract

**Background:**

Post-endoscopic retrograde cholangiopancreatography (ERCP) pancreatitis (PEP) is one most frequent and severe complication of ERCP. In consideration of recent advancements in both endoscopic and artificial intelligence research, it is possible to construct a practical risk prediction model to facilitate the identification of PEP patients at elevated risk.

**Aim:**

We developed and validated a concise predictive model for post-ERCP pancreatitis risk with logistic regression (LR), LightGBM, Support Vector Machine (SVM), XGBoost, and Multilayer Perceptron (MLP) neural network models.

**Methods:**

We selected 688 patients undergone ERCP to form the basic dataset, with 70% for training and 30% for validation. Subsequently, Stepwise Backward Selection Based on Logistic Regression was utilized to select pertinent clinical features, incorporating the machine learning (ML) models to construct the final predictive model. The efficacy of the model was evaluated by various metrics. These newly identified clinical features were then incorporated into a simplified, points-based risk scoring system for potential bedside application and further evaluation.

**Results:**

Based on the collected data and the results of stepwise backward regression, we identified the following features as potentially significant clinical variables that influence the risk of post-ERCP pancreatitis: periampullary diverticulum, pancreatic stent placement, pancreatic guidewire passages, dilation of the extrahepatic bile duct, age, and coronary artery disease, and constructed a prediction model. Following this, several ML models were constructed to assess the performance of this model. All ML models demonstrated superior performance to conventional logistic regression (LR) models in terms of AUC curves, with XGBoost, SVM, LightGBM, and MLP models all achieving at least acceptable performance levels. Finally, we developed a simplified scoring system based on LightGBM model with an AUC of 0.75.

**Conclusions:**

We developed and validated a concise predictive model for post-ERCP pancreatitis risk, and a simplified scoring system based on the LightGBM model. This model facilitates individual risk prediction and preventive strategy selection.

## Introduction

Post-endoscopic retrograde cholangiopancreatography (ERCP) pancreatitis (PEP) is one most frequent and serious complication following ERCP, with an incidence rate from 2.1% to 15.1% (1). Although most PEP cases are mild or moderate, specific conditions can significantly extend hospitalization for patients and be fatal in severe instances (2). Based on these concerns, multitude of risk prediction models for PEP have been suggested, incorporating various patient and procedure-related factors such as gender, difficult cannulation, history of pancreatitis, pancreatic duct cannulation, etc. (3). However, due to the intricate interplay of risk factors that may even synergize, these models often suffer from limited discriminative power, complexity, or lack of external validation, making their limited application in clinical practice.

In recent years, machine learning (ML) has gained considerable attention in clinical medical settings. ML algorithms analyze a wealth of variables with complex relationships using methods such as supervised, unsupervised, and semi-supervised learning, offering advantages such as intuitiveness and high predictive efficiency (4). Research indicates that computer-aided diagnostic models substantially assist clinicians in diagnosing and predicting diseases (4). At present, many ML models and algorithms based on diverse architectures have been developed, showing impressive performance in predicting significant diseases in the medical field (5). Hence, our goal is to develop and validate practical PEP prediction models using the latest ERCP database from General Hospital of Ningxia Medical University. This study pursues two objectives: (1) to compare the performance of different ML models in predicting PEP with stringent inclusion and exclusion criteria; (2) to identify a clinically relevant model with as few predictive factors as possible through innovative approaches, aiding endoscopists in decision-making and planning postoperative management.

## Materials and methods

### Patient characteristics

**Figure 1:**
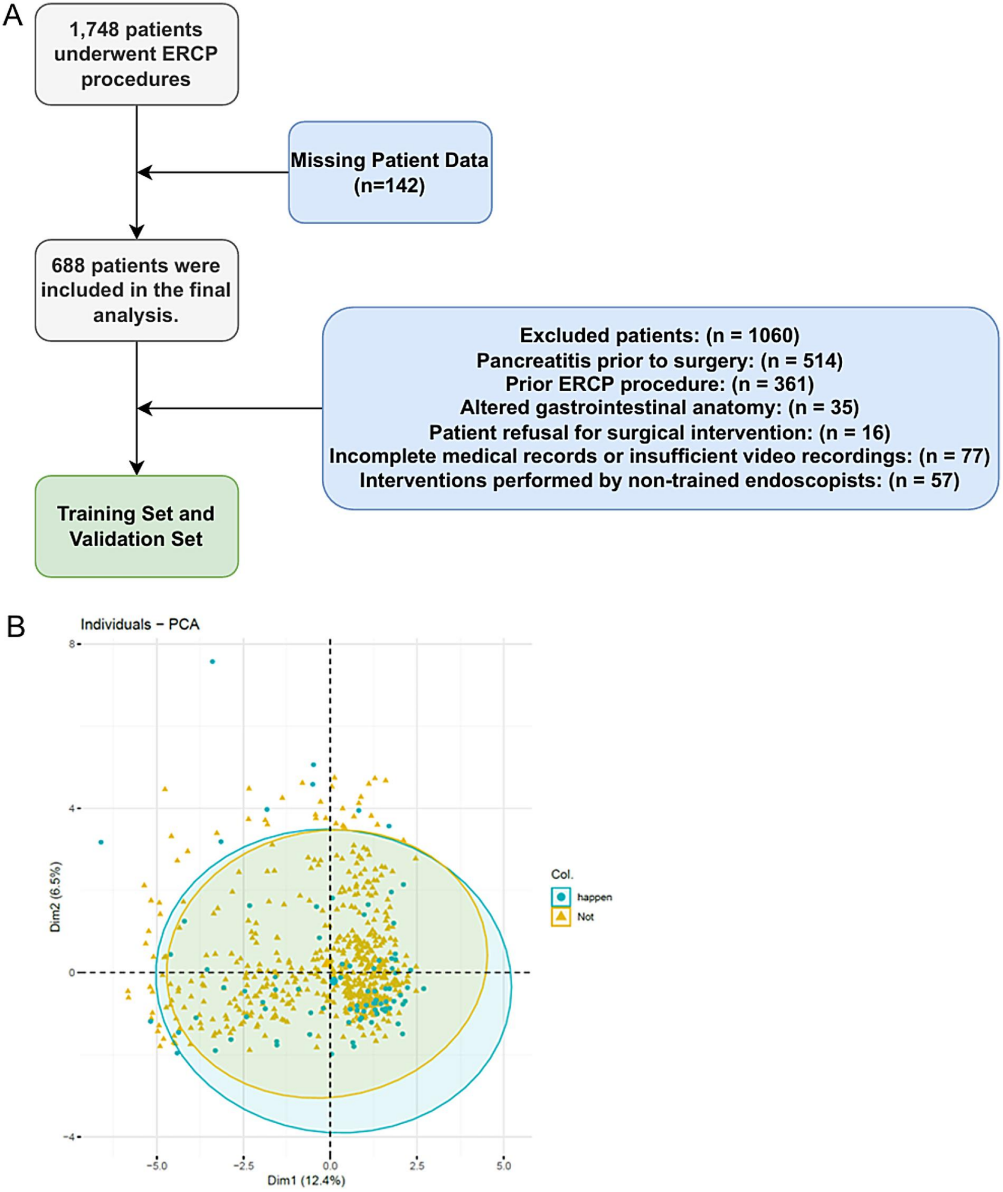
The pipeline and criteria for ERCP patient selection and exclusion in this study **(A)** and the PCA plot for ADASYN **(B)**.

We conducted a retrospective analysis of patients' clinical data from diagnostic or therapeutic ERCPs at General Hospital of Ningxia Medical University from May 2022 to June 2023. After applying rigorous inclusion and exclusion criteria, we included a total of 688 cases in the training and validation sets. Eligible patients were those with relevant biliary-pancreatic diseases indicated for ERCP, who had provided written informed consent for the procedure and had granted verbal or written permission for postoperative examinations. Patients were excluded if they presented with acute pancreatitis, had a history of previous ERCP or gastrointestinal reconstructive surgery. Patients that were under 18 years-old (6), had incomplete medical records or surgical videos, did not complete necessary follow-up examinations in a timely manner, did not undergo a full cannulation attempt and abandoned the surgery, or were treated by an endoscopist with less than 50 cannulate procedures (7), were also excluded (Figure 1).

### Study endpoint and definitions of outcomes

The primary endpoint was established according to current international guidelines and consensus on the incidence of PEP. The criteria for the occurrence of PEP were defined as follows: (1) New onset of abdominal pain was consistent with pancreatitis (acute persistent upper left abdominal pain); (2) Serum amylase levels were three times greater than the upper limit of normal within 24 h after the procedure; (3) Imaging evidence of pancreatitis such as peripancreatic fluid extravasation, pancreatic gland enlargement due to edema, pancreatic duct dilation, pancreatic tissue necrosis, or formation of a pancreatic pseudocyst (two out of these three criteria must be met by the Atlanta Consensus 2012) (7, 8). Failed cannulation was defined as the inability to correctly enter the bile or pancreatic duct despite all techniques and efforts (9). Difficult cannulation was characterized by a cannulation time exceeding 5 min, more than five papillary contacts, or incorrect entry into the pancreatic duct more than once by ESGE Guidelines 2019 (2, 10). We further monitored preoperative serum calcium ion levels, classifying them using our hospital's upper limit of normal value at 2.12 mmol. In this study, the threshold of patient's age was set at <60. The biliary brush cytology sampling methods included cytological brushing or forceps biopsy (11). “Failure to clear bile duct stones” was determined as stones that were not retrieved or not completely retrieved following the full cannulation process. The patient retained a nasobiliary drain at the conclusion of the procedure (12).

### Data processing

To ensure the stability of the results, patients with missing video recording data were excluded (*n* = 142), which did not affect the results of this study. As the incidence of patients with PEP at our institution was approximately 11%, there was an imbalance in the proportion of patients with and without PEP. To address the imbalance in sample sizes at different levels of the outcome variable, we used the Adaptive Synthetic Sampling (ADASYN) algorithm. This approach was used to survey patients with PEP and normal samples accordingly, achieving a 1:1 match with patients who did not develop PEP postoperatively (13).

### Data separation and feature filtering

Prior to oversampling with ADASYN, we performed data separation by randomly dividing the dataset into two subsets (training and test) with a 7:3 ratio. These subsets were used for modelling and validation, respectively. To minimize the overfitting risk introduced by ADASYN oversampling, we implemented a robust validation strategy. Specifically, we employed 10-fold cross-validation during the model training and tuning phase. Furthermore, the final model was evaluated on a completely independent test set that was not involved in either the oversampling or cross-validation processes. Feature selection within the training set was conducted by a stepwise regression approach grounded in logistic regression with backward elimination (14). Covariance test showed no significant abnormality. This procedure was implemented using the Mass package in R software (version 4.2.2). Variables were selected according to the principle of minimising the Akaike Information Criterion (AIC) and a threshold of *p*-value < 0.01. This process aimed to identify a subset of features that provided an optimal balance between power and dimensionality (15).

### ML model building, evaluation and interpretation

The dataset was subjected to both linear and non-linear ML models, including logistic regression (LR), support vector machines (SVM), gradient boosting trees (GBT) and neural network models. The SVM was implemented using both Linear and Radial Basis Function (RBF) kernels, the GBT was implemented using XGBoost (eXtreme Gradient Boosting) and LightGBM (Light Gradient Boosting Machine), while the neural network model was implemented using a Multi-Layer Perceptron (MLP). The predictive performance of all models was evaluated using five metrics: Area Under the Receiver Operating Characteristic Curve (AUROC), precision, recall, F1 score and accuracy. Finally, ML models with acceptable performance were selected based on the AUC curve.

### Statistical analyses

Categorical variables were assigned numerical codes, and categorical variables were assigned numerical codes, and ization was conducted using EXCEL 2018. Python was used for data processing and model development, relying on the scikit-learn library for the construction of machine learning algorithms. Data statistics, variable selection and construction of conventional LR models were performed using R software (R4.2.2), with a *p*-value < 0.01 indicating statistical significance. All authors of this article had access to the research data, participated in data collection, reviewed and approved the final manuscript.

## Results

### Study population and baseline characteristics

A total of 1,748 patients who underwent ERCP procedures met the criteria for this study. Of these, 514 patients with pre-operative acute pancreatitis (hyperamylasemia) were excluded. Further 396 patients with previous ERCP or gastrointestinal reconstruction history were also excluded. A further 150 patients were excluded because the procedure was abandoned or performed by inexperienced endoscopist, the medical records or video data were incomplete. Each exclusion criterion was counted only once. Therefore, 688 patients were included in the final analysis. The study flow chart was shown in Figure 1. Table 1 showed the baseline characteristics of the study population, focusing on the variable PEP. The distribution of key characteristics, including sex, age, presence of diabetes, coronary heart disease, hypertension, history of biliary surgery, cholecystectomy, acute pancreatitis, Ca + and total bilirubin levels, and various clinical procedures and interventions, were reported. We then performed the PCA dimensionality reduction plot for ADASYN and found on difference between PEP (happen) and non-PEP (Not) patients (Figure 1B).

**Table 1:** The characteristics and their values considered in this study.

Characteristic	PEP	
	Y, *N* = 88^a^	N, *N* = 600^a^
Sex		
Male	45 (51.1%)	327 (54.5%)
Female	43 (48.9%)	273 (45.5%)
Age		
>60	41 (46.6%)	392 (65.3%)
<60	47 (53.4%)	208 (34.7%)
Diabetes		
N	76 (86.4%)	517 (86.2%)
Y	12 (13.6%)	83 (13.8%)
Coronary heart disease		
N	81 (92.0%)	535 (89.2%)
Y	7 (8.0%)	65 (10.8%)
Hypertension		
N	68 (77.3%)	452 (75.3%)
Y	20 (22.7%)	148 (24.7%)
History of biliary surgery		
N	79 (89.8%)	556 (92.7%)
Y	9 (10.2%)	44 (7.3%)
History of cholecystectomy		
N	80 (90.9%)	542 (90.3%)
Y	8 (9.1%)	58 (9.7%)
History of acute pancreatitis		
Y	10 (11.4%)	33 (5.5%)
N	78 (88.6%)	567 (94.5%)
Ca + (mmol/L)		
>22.1	40 (45.5%)	307 (51.2%)
<22.1	48 (54.5%)	293 (48.8%)
Total bilirubin level (umol/L)		
<22 umol/L	69 (78.4%)	453 (75.5%)
≥22 umol/L	19 (21.6%)	147 (24.5%)
Dilatation of extrahepatic bile ducts		
Y	65 (73.9%)	503 (83.8%)
N	23 (26.1%)	97 (16.2%)
Direct bilirubin level (umol/L)		
<5 umol/L	34 (38.6%)	249 (41.5%)
≥5 umol/L	54 (61.4%)	351 (58.5%)
Gallbladder stone		
N	64 (72.7%)	433 (72.2%)
Y	24 (27.3%)	167 (27.8%)
Common bile duct stones		
Y	61 (69.3%)	422 (70.3%)
N	27 (30.7%)	178 (29.7%)
Acute cholangitis		
Y	67 (76.1%)	432 (72.0%)
N	21 (23.9%)	168 (28.0%)
Benign biliary stricture		
N	82 (93.2%)	569 (94.8%)
Y	6 (6.8%)	31 (5.2%)
Malignancy biliary stricture		
N	73 (83.0%)	523 (87.2%)
Y	15 (17.0%)	77 (12.8%)
Malignant diseases of the pancreas		
N	80 (90.9%)	527 (87.8%)
Y	8 (9.1%)	73 (12.2%)
pancreatic duct calculus		
N	87 (98.9%)	597 (99.5%)
Y	1 (1.1%)	3 (0.5%)
Mechanical stone crushing		
N	85 (96.6%)	589 (98.2%)
Y	3 (3.4%)	11 (1.8%)
Stone residue		
N	9 (10.2%)	27 (4.5%)
Y	79 (89.8%)	573 (95.5%)
Total duration of cannulation		
≥5min	23 (26.1%)	94 (15.7%)
<5min	65 (73.9%)	506 (84.3%)
Total number of cannulation		
≥5 times	24 (27.3%)	73 (12.2%)
<5 times	64 (72.7%)	527 (87.8%)
Number of guidewire entries into the pancreatic duct		
Pancreatic guidewire passages ≤1	66 (75.0%)	545 (90.8%)
Pancreatic guidewire passages >1	22 (25.0%)	55 (9.2%)
Pancreatic injection		
N	72 (81.8%)	556 (92.7%)
Y	16 (18.2%)	44 (7.3%)
Pancreatic stent placement		
N	82 (93.2%)	524 (87.3%)
Y	6 (6.8%)	76 (12.7%)
Endoscopic biliary stenting		
N	74 (84.1%)	521 (86.8%)
Y	14 (15.9%)	79 (13.2%)
EST		
Y	77 (87.5%)	540 (90.0%)
N	11 (12.5%)	60 (10.0%)
Endoscopic nasobiliary drainage		
Y	78 (88.6%)	537 (89.5%)
N	10 (11.4%)	63 (10.5%)
Sampling from the biliary tract		
N	82 (93.2%)	582 (97.0%)
Y	6 (6.8%)	18 (3.0%)
Periampullary diverticulum		
N	40 (45.5%)	466 (77.7%)
Y	48 (54.5%)	134 (22.3%)
Balloon dilatation		
N	62 (70.5%)	450 (75.0%)
Y	26 (29.5%)	150 (25.0%)

a *n* (%).

### Feature filtering and prediction model construction

Based on the characteristic sets listed in Table 1, we used a stepwise regression method with logistic regression and backward elimination to select variables for feature filtering. Seven characteristic sets were included in the features with smaller AIC curves in the training set and a *p*-value < 0.01: Periampullary diverticulum (PAD), pancreatic stent placement (PSP), Pancreatic injection (PI), number of guidewire passages into the pancreatic duct (NGP), dilatation of extrahepatic bile ducts (DEBD), age, and coronary heart disease (CHD). Based on the logistic regression analysis results table (Tables 2–4), the conventional LR model predicts the probability of pancreatitis using the following equation:

$$\begin{array}{rl} & P=\exp\;(0.154+1.767\;*\;\mathrm{PAD}-0.624\;*\;\mathrm{PSP};+0.567\;*\;\mathrm{PI},\\ & \;+\;1.075\;*\;\mathrm{NGP}-0.383\;*\;\mathrm{DEBD}-0.639\;*\;\mathrm{CHD}\\ & \;-1.176\;*\;\mathrm{Age})/(1+\;\;\exp(0.154+1.767\;*\;\mathrm{PAD}\;-0.624\;*\;\mathrm{PST}\\ & \;+\;0.567\;*\;\mathrm{PI}+1.075\;*\;\;\mathrm{NGP}-0.383\;*\;\mathrm{DEBD}-0.639\;*\;\mathrm{CHD}\\ & \;-1.176\;*\;\mathrm{Age})) \end{array}$$

**Table 2:** Logistic regression with stepwise variable reduction.

Dependent: PEP	Value	OR (univariable)	OR (multivariable)
History of cholecystectomy	N	–	–
	Y	0.55 (0.35–0.85, *p* = 0.008)	0.63 (0.36–1.08, *p* = 0.097)
Balloon dilatation	N	–	–
	Y	1.04 (0.81–1.35, *p* = 0.744)	1.35 (0.97–1.87, *p* = 0.072)
EST	N	–	–
	Y	0.81 (0.56–1.16, *p* = 0.246)	0.63 (0.39–1.00, *p* = 0.051)
Periampullary diverticulum	N	–	–
Endoscopic biliary stenting	Y	4.26 (3.33–5.48, *p* < 0.001)	7.06 (5.16–9.76, *p* < 0.001)
–	N	-	–
	Y	1.05 (0.75–1.46, *p* = 0.776)	–
Pancreatic duct calculus	N	–	–
	Y	0.98 (0.18–5.31, *p* = 0.981)	–
Sampling from the biliary tract	N	–	–
Pancreatic stent placement	Y	1.78 (1.00–3.28, *p* = 0.054)	4.08 (2.01–8.53, *p* < 0.001)
	N	–	–
	Y	0.38 (0.24–0.58, *p* < 0.001)	0.40 (0.23–0.67, *p* = 0.001)
Pancreatic injection	N	–	–
	Y	1.82 (1.24–2.70, *p* = 0.003)	1.70 (1.04–2.80, *p* = 0.036)
Number of guidewire entries into the pancreatic duct	Pancreatic guidewire passages ≤1	–	–
	Pancreatic guidewire passages >1	2.99 (2.15–4.21, *p* < 0.001)	3.92 (2.58–6.01, *p* < 0.001)
Total number of cannulations	≥5 times	–	–
	<5 times	1.95 (1.43–2.67, *p* < 0.001)	–
Total duration of cannulation	≥5min	-	–
	<5min	1.66 (1.24–2.22, *p* = 0.001)	1.34 (0.94–1.93, *p* = 0.109)
Stone residue	N	–	–
	Y	1.97 (1.23–3.22, *p* = 0.006)	2.20 (1.21–4.07, *p* = 0.010)
Dilatation of extrahepatic bile ducts	Nondilated extrahepatic bile duct	1.80 (1.36–2.40, *p* < 0.001)	1.85 (1.30–2.65, *p* = 0.001)
	Dilatation of extrahepatic bile ducts	–	–
Malignancy biliary stricture	N	–	–
	Y	1.37 (1.00–1.90, *p* = 0.051)	2.34 (1.44–3.84, *p* = 0.001)
Malignant diseases of the pancreas	N	–	–
	Y	0.61 (0.42–0.90, *p* = 0.013)	–
Benign biliary stricture	N	–	–
	Y	0.81 (0.47–1.39, *p* = 0.451)	0.52 (0.26–1.02, *p* = 0.057)
Acute cholangitis	N	–	–
	Y	1.28 (0.99–1.65, *p* = 0.065)	3.74 (2.01–7.08, *p* < 0.001)
Gallbladder stone	N	–	–
	Y	0.91 (0.71–1.17, *p* = 0.467)	0.78 (0.57–1.07, *p* = 0.129)
Mechanical stone crushing	N	–	–
	Y	0.98 (0.42–2.31, *p* = 0.963)	–
Endoscopic nasobiliary drainage	N	–	–
	Y	1.16 (0.80–1.70, *p* = 0.430)	–
Total bilirubin level (umol/L)	<22 umol/L	–	–
	≥22 umol/L	1.45 (1.10–1.91, *p* = 0.009)	–
History of acute pancreatitis	N	–	–
	Y	1.53 (0.97–2.43, *p* = 0.067)	1.87 (1.07–3.30, *p* = 0.029)
Direct bilirubin level (umol/L)	<5 umol/L	–	–
	≥5 umol/L	1.21 (0.96–1.53, *p* = 0.103)	1.31 (0.98–1.75, *p* = 0.068)
Common bile duct stones	N	–	–
	Y	1.04 (0.82–1.34, *p* = 0.728)	0.33 (0.18–0.59, *p* < 0.001)
History of biliary surgery	N	–	–
	Y	0.98 (0.63–1.51, *p* = 0.923)	–
Sex	Male	–	–
	Female	0.83 (0.66–1.04, *p* = 0.104)	-
Diabetes	N	–	–
	Y	0.82 (0.58–1.15, *p* = 0.244)	–
Age	>60	–	–
	<60	0.43 (0.34–0.54, *p* < 0.001)	0.29 (0.22–0.39, *p* < 0.001)
Hypertension	N	–	–
	Y	0.65 (0.49–0.86, *p* = 0.003)	–
Coronary heart disease	N	–	–
	Y	0.54 (0.36–0.82, *p* = 0.004)	0.44 (0.26–0.75, *p* = 0.003)
Ca + (mmol/L)	>22.1	–	–
	<22.1	0.78 (0.62–0.98, *p* = 0.030)	0.73 (0.55–0.96, *p* = 0.024)

**Table 3:** Logistic regression with stepwise variable reduction.

Step Df	Deviance	Resid. Df	Resid. Dev	AIC
Sex	0.002127310	1,180	1,282.551	1,346.551
Mechanical stone crushing	0.007754036	1,181	1,282.559	1,344.559
Endoscopic biliary stenting	0.077781737	1,282	1,282.637	1,342.637
Total bilirubin level	0.110931218	1,183	1,282.747	1,340.747
pancreatic duct calculus	0.593770974	1,184	1,283.341	1,339.341
Malignant diseases of the pancreas	0.476935189	1,185	1,283.818	1,337.818
Total number of cannulations	0.476935189	1,186	1,284.450	1,336.450
History of biliary surgery	0.568842898	1,187	1,285.019	1,335.019
Hypertension	0.843645419	1,188	1,286.421	1,334.421
Diabetes	0.843645419	1,189	1,287.265	1,333.265
Endoscopic nasobiliary drainage	1.511438520	1,190	1,288.776	1,332.77

**Table 4:** Covariance diagnostics.

Term	VIF	VIFCI_low	VIF_CI_high	Tolerance	Tolerance_CI_low	Tolerance_CI_high	SE_factor
Sex	1.221	1.155	1*.316*	1.105	0.819	0.760	0.866
Age	1.231	1.163	1.326	1.109	0.812	0.754	0.860
Diabetes	1.856	1.720	2.018	1.362	0.539	0.496	0.581
coronary heart disease	1.702	1.582	1.847	1.305	0.587	0.541	0.632
Hypertension	1.504	1.405	1.627	1.226	0.665	0.615	0.712
History of biliary surgery	1.168	1.109	1.259	1.081	0.856	0.794	0.902
History of cholecystectomy	1.110	1.060	1.202	0.900	0.832	0.943	1.054
History of acute pancreatitis	1.092	1.045	1.186	0.915	0.843	0.956	1.045
Ca + (mmol/L)	1.092	1.046	1.186	1.045	0.915	0.843	0.956
Total bilirubin level (umol/L) Dilatation of	1.761	1.635	1.913	1.327	0.568	0.523	0.612
Extrahepatic bile ducts	1.138	1.084	1.229	1.067	0.878	0.813	0.923
Direct bilirubin level(umol/L)	1.874	1.736	2.038	1.369	0.534	0.491	0.576
Gallbladder stone	1.179	1.118	1.271	1.086	0.848	0.787	0.894
Common bile duct stones	2.682	2.460	2.939	1.638	0.373	0.340	0.407
Acute cholangitis	3.525	3.215	3.879	1.878	0.284	0.258	0.311
Benign biliary stricture	1.330	1.251	1.435	1.153	0.752	0.697	0.799
Malignancy biliary stricture	2.784	2.551	3.052	1.668	0.359	0.328	0.392
Malignant diseases of the pancreas	2.014	1.861	2.193	1.419	0.497	0.456	0.537
pancreatic duct calculus	1.130	1.077	1.221	1.063	0.885	0.819	0.929
Mechanical stone crushing	1.000	1.000	Inf	1.000	1.000	0.000	1.000
Stone residue	1.283	1.209	1.383	1.133	0.779	0.723	0.827
Total duration of cannulation	1.124	1.071	1.215	1.060	0.890	0.823	0.933
Total number of cannulations	1.300	1.224	1.401	1.140	0.769	0.714	0.817
Number of guidewire entries into the pancreatic duc	1.275	1.202	1.374	1.129	0.784	0.728	0.832
Pancreatic injection	1.336	1.256	1.441	1.156	0.749	0.694	0.796
Pancreatic stent placement	1.279	1.205	1.378	1.131	0.782	0.726	0.830
Endoscopic biliary stenting	2.975	2.722	3.265	1.725	0.336	0.306	0.367
EST	1.452	1.359	1.569	1.205	0.689	0.637	0.736
Endoscopic nasobiliary drainage	2.624	2.407	2.874	1.620	0.381	0.348	0.415
Sampling from the biliary tract	1.573	1.466	1.703	1.254	0.636	0.587	0.682
Periampullary diverticulum	1.243	1.174	1.339	1.115	0.805	0.747	0.852
Balloon dilatation	1.255	1.185	1.353	1.120	0.797	0.739	0.844

**Figure 2:**
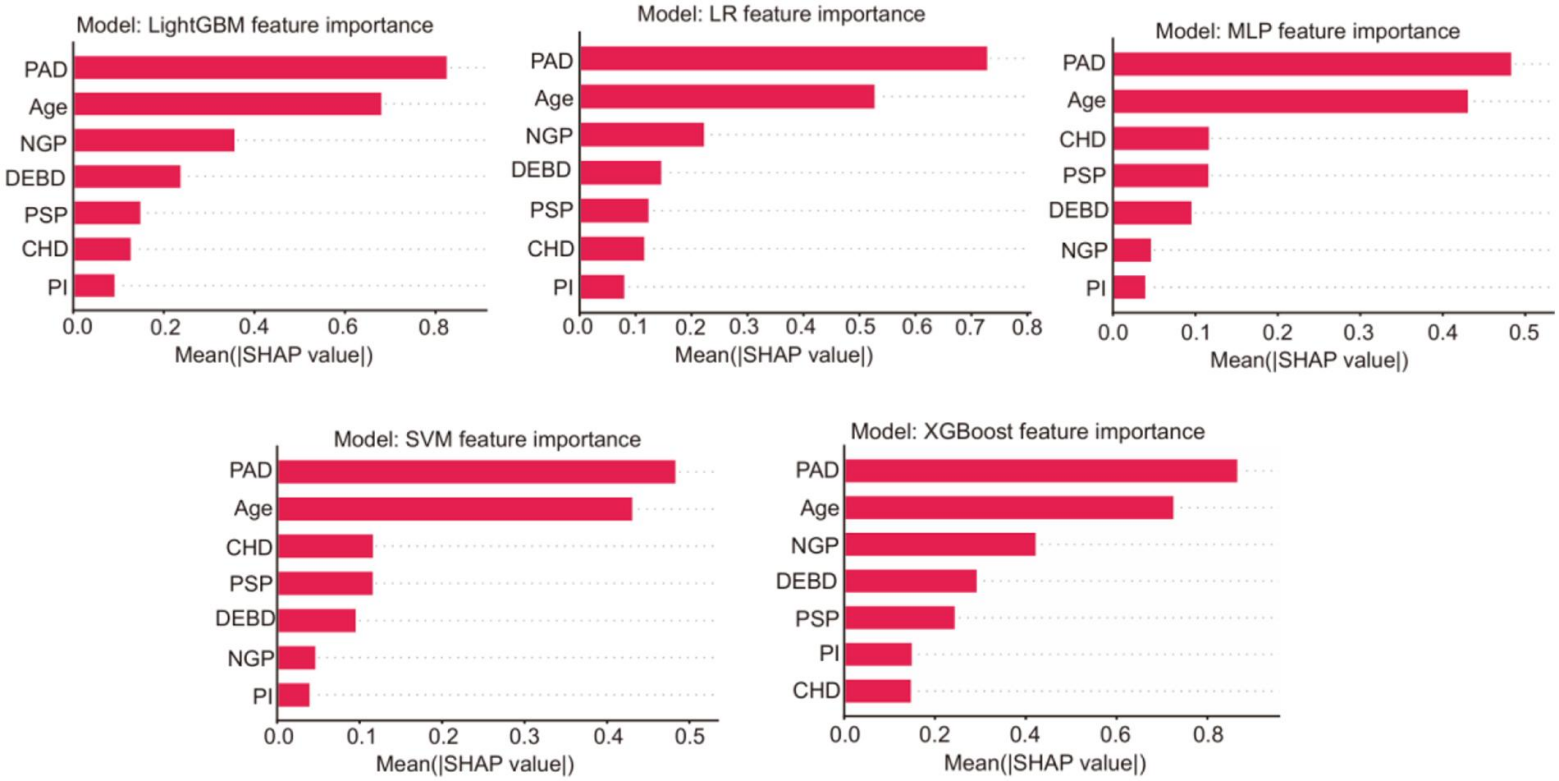
Bar plot showing the absolute mean SHAP values for the seven features in each model.

To enhance the interpretability and clinical reliability of our model, we conducted a comprehensive Shapley additive explanations (SHAP) analysis and calibration analysis. For the five models, the SHAP summary figures intuitively showed the contribution of each feature to the model prediction, and highlights the most influential clinical variables (Figure 2). After that, we chose the intersection features selected by LASSO regression and stepwise methods for modeling, and finally removed the pancreatic injection (PI) feature from the equation. The final prediction equation was:

$$\begin{array}{rl} & P\;=\;\exp(-0.226\;+\;1.739\;*\;\mathrm{PAD}\;-0.617\;*\;\mathrm{PSP}\;+\;1.235\;*\;\mathrm{NGP}\;\\ & \;\;\;+\;0.43\;*\;\mathrm{DEBD}\;-\;0.601\;*\;\mathrm{CHD}\;-\;1.128\;*\;\mathrm{Age})/\\ & \;\;\;\;(1\;+\;\exp(-0.226\;+\;1.739\;*\;\mathrm{PAD}\;-\;0.617\;*\;\mathrm{PSP}\;\\ & \;\;\;+\;1.235\;*\;\mathrm{NGP}+0.43\;*\;\mathrm{DEBD}-0.601\;*\;\mathrm{CHD}\\ & \;\;\;-\;1.128\;*\;\mathrm{Age})) \end{array}$$

At the same time, we considered the CHD patients taking aspirin. Among 72 patients diagnosed with CHD, 50 patients (69.4%) regularly took aspirin before ERCP surgery, and only one patient (2.0%) had PEP incidence. For the 22 non-aspirin group, the PEP incidence rate was 18.2% (4/22), showing significant difference compared with aspirin-taken group (*p*-value = 0.024), indicating that aspirin effect on PEP incidence for CHD patients.

### Model performance evaluation

**Figure 3:**
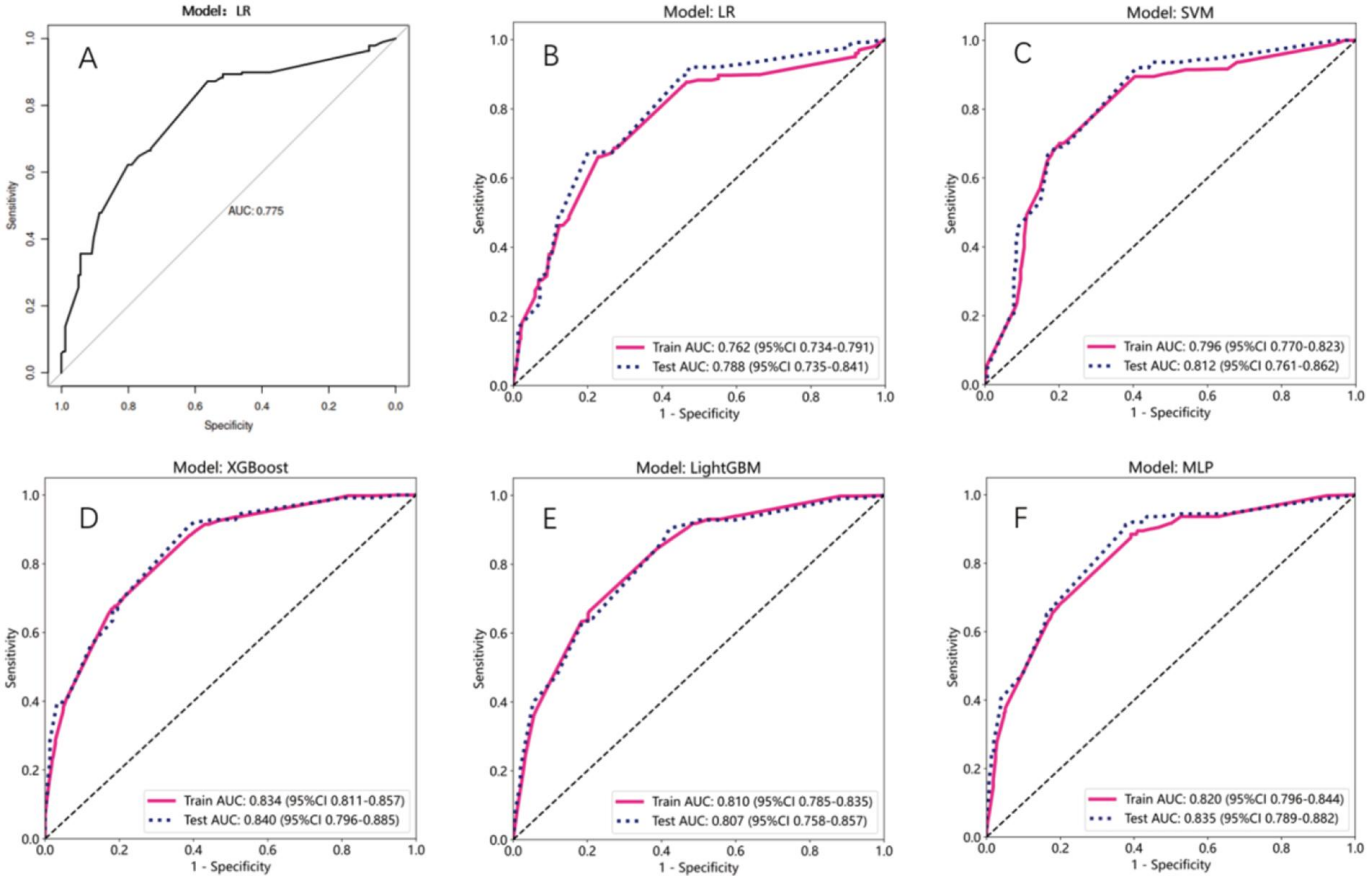
Assessment of the AUC values for multiple models. **(A–F)** Line plot represented the ROC curves for different models. *X*-axis is specificity, and *Y*-axis is sensitivity. Red solid line and blue dashed line represented train and test AUC, respectively.

The predictive performance that was measured by the receiver operating characteristic (ROC) model by comparing to the conventional logistic regression (LR) model, was depicted (Figures 3A–F). Models with an area under the ROC curve (AUC) exceeding 0.75 were considered as acceptable. This criterion was met by the following models: LR (Figure 3A) with a test AUC of 0.775 [95% confidence interval (CI) 0.727–0.823]; LR (ML) (Figure 3B) with a test AUC of 0.788 (95% CI 0.7347–0.8408); support vector machine (SVM) (Figure 3C) with a test AUC of 0.812 (95% CI 0.7612–0.8624); XGBoost (Figure 3D) with a test AUC of 0.840 (95% CI 0.7955–0.8851); LightGBM (Figure 3E) with a test AUC of 0.807 (95% CI 0.7575–0.8574); and the multilayer perceptron (MLP) model (Figure 3F) with a test AUC of 0.835 (95% CI 0.7887–0.8822). A detailed performance description for each ML model is supplemented in Table 5.

**Table 5:** The performance of all ML models.

model name	Accuracy	AUC	95% CI	Sensitivity	Specificity	PPV	NPV	Precision	Recall	F1	Threshold	Task
LR	0.687	0.762	0.7336–0.7905	0.489	0.850	0.729	0.669	0.729	0.489	0.585	0.518	PEP-train
LR	0.707	0.788	0.7347–0.8408	0.516	0.864	0.756	0.686	0.756	0.516	0.613	0.518	PEP-test
SVM	0.754	0.796	0.7696–0.8230	0.697	0.801	0.743	0.762	0.743	0.697	0.719	0.430	PEP-train
SVM	0.743	0.812	0.7612–0.8624	0.698	0.779	0.721	0.759	0.721	0.698	0.710	0.246	PEP-test
XGBoost	0.750	0.834	0.8110–0.8567	0.701	0.790	0.733	0.762	0.733	0.701	0.717	0.451	PEP-train
XGBoost	0.750	0.840	0.7955–0.8851	0.690	0.799	0.737	0.759	0.737	0.690	0.713	0.451	PEP-test
LightGBM	0.734	0.810	0.7852–0.8346	0.657	0.798	0.728	0.739	0.728	0.657	0.691	0.468	PEP-train
LightGBM	0.718	0.807	0.7575–0.8574	0.865	0.597	0.637	0.844	0.637	0.865	0.734	0.406	PEP-test
MLP	0.729	0.820	0.7957–0.8444	0.875	0.608	0.648	0.856	0.648	0.875	0.745	0.430	PEP-train
MLP	0.750	0.835	0.7887–0.8822	0.905	0.623	0.663	0.889	0.663	0.905	0.765	0.430	PEP-test

**Figure 4:**
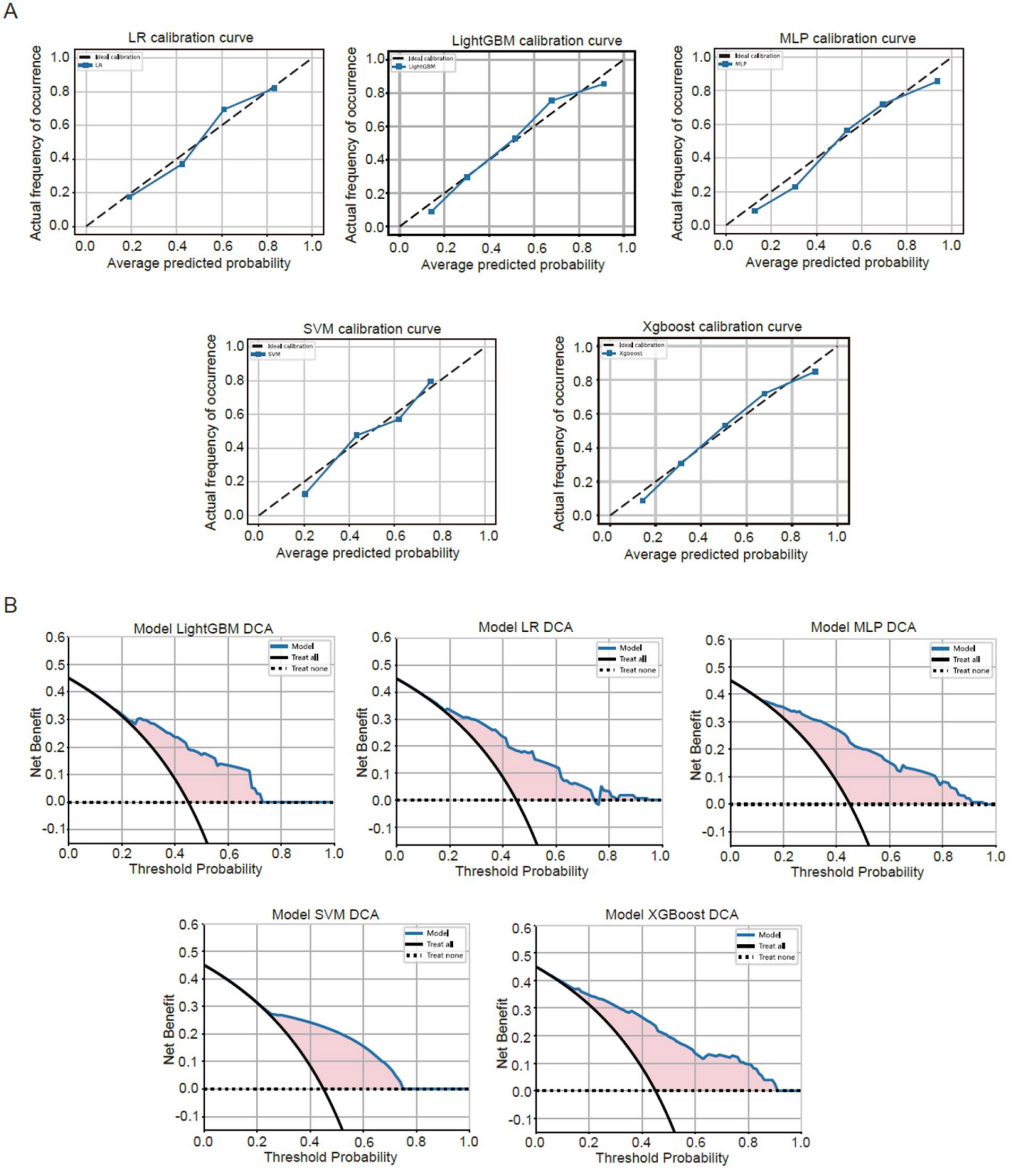
Assessment of the five models. **(A)** Line plot showing the calibration analysis results for each model. **(B)** Line plot showing the decision-curve analysis results to evaluate clinical benefit for each model.

Then we performed calibration analysis for each model, the calibration curves indicated that Logistic Regression (LR) demonstrated nearly ideal calibration, while LightGBM and Xgboost also showed reasonable calibration performance. In contrast, the calibration curves for SVM and MLP exhibit minor deviations from the ideal line, which remain within acceptable limits and do not substantially affect the clinical interpretability of the predictions (Figure 4A). Finally, we included a decision-curve analysis (DCA) to evaluate clinical benefit at different thresholds. This analysis allows for a direct comparison of the net clinical benefit across a range of threshold probabilities, providing a crucial perspective on the model's value in clinical decision-making beyond traditional discrimination metrics (Figure 4B).

**Figure 5:**
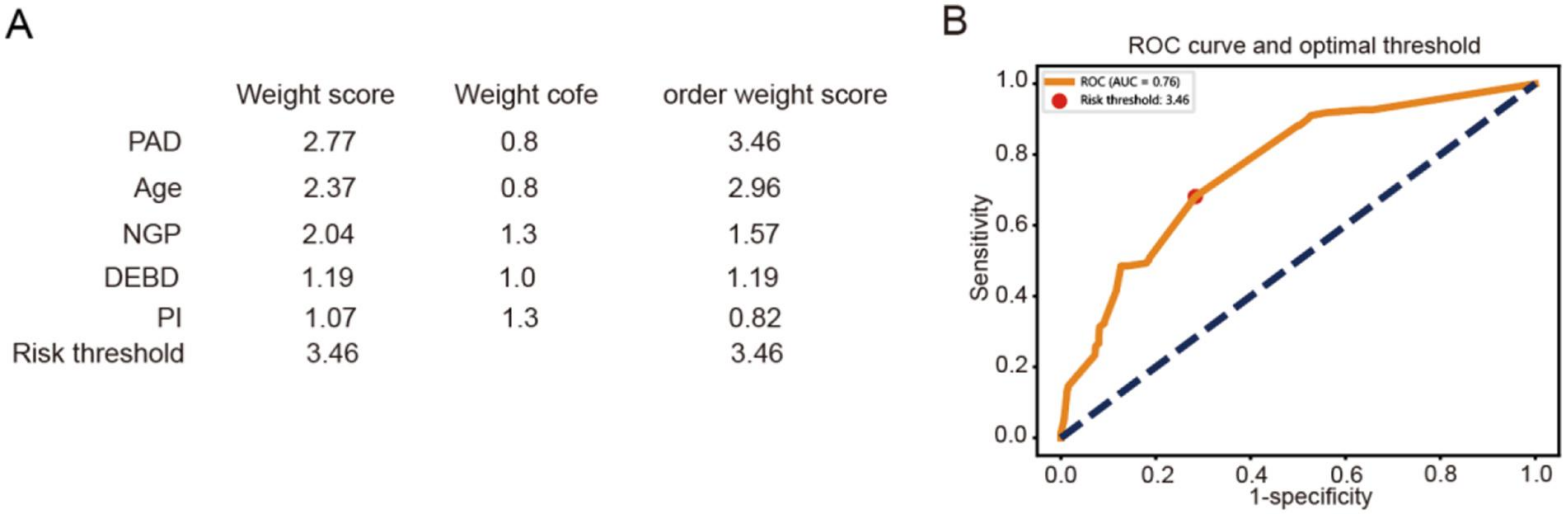
Construction of a simplified, points-based risk scoring system. **(A)** The points-based risk scoring system for potential bedside application. **(B)** Line plot represented the ROC curve for the system.

Finally, we developed a simplified, points-based risk scoring system for potential bedside application based on the relative SHAP importance using the LightGBM model, which showed higher AUC score than other models. Using a pre-specified threshold of 3.46 points (derived from the risk distribution in our cohort), patients can be stratified into a Low-Risk group (score ≤3.46) and a High-Risk group (score >3.46) (Figure 5A). This simplified model, despite its ease of use, retained a clinically acceptable discriminative ability with an AUC of 0.75 on our validation set (Figure 5B).

## Discussion

### Major findings

**Figure 6:**
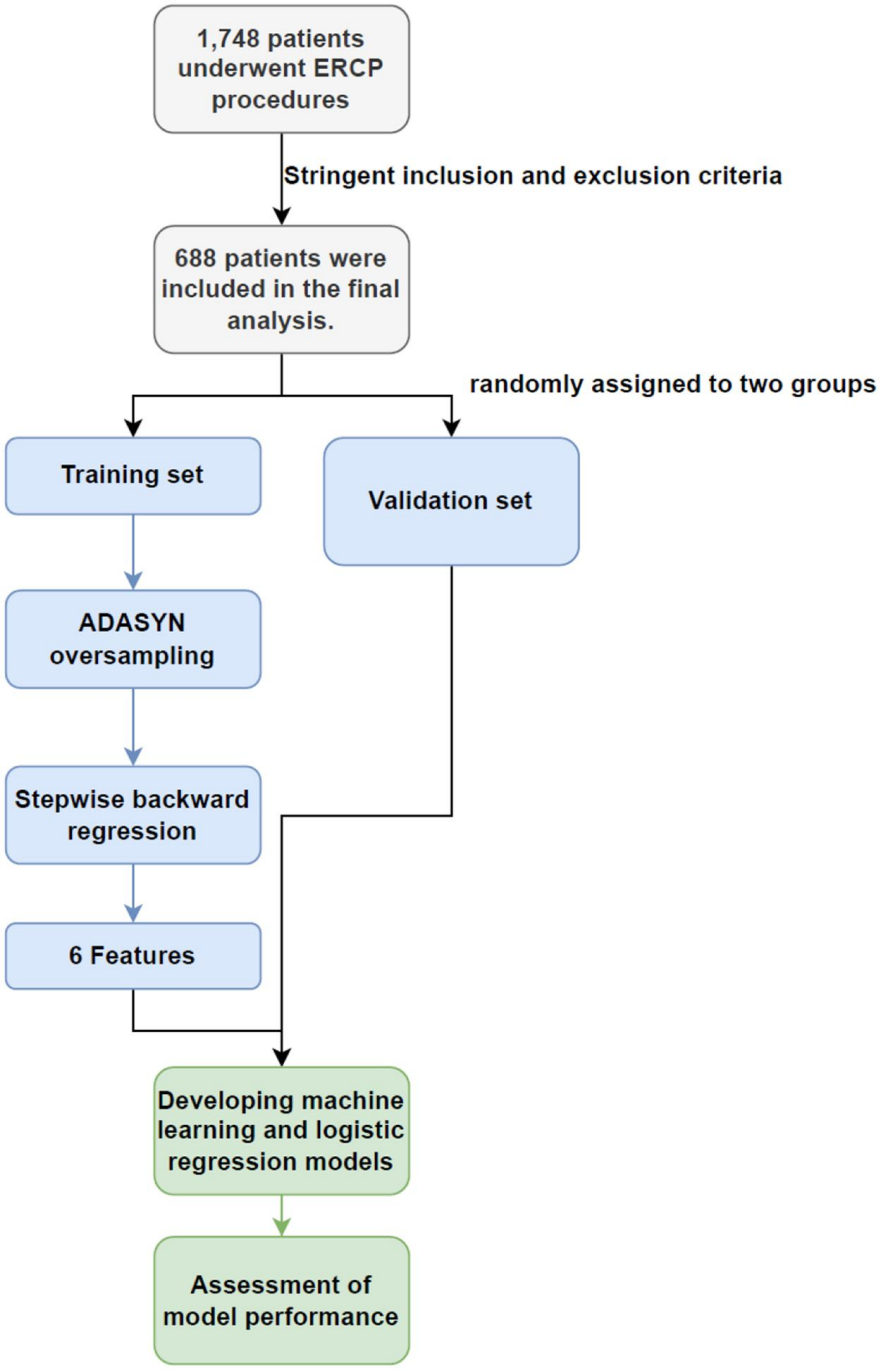
The ERCP patient selection and model construction pipeline in this study.

In this study, we developed a predictive model for post-ERCP pancreatitis using ML techniques, employing stepwise regression for feature selection. Based on stringent inclusion and exclusion criteria, we independently identified six factors showing highest correlation with outcomes. We consider periampullary diverticulum, pancreatic duct stent placement, more than one guidewire passage into the pancreatic duct, non-dilated extrahepatic bile duct, age, and coronary heart disease as the most significant clinical variables affecting the risk of PEP. In subsequent ML model development, all ML models outperformed the conventional LR model, with Support Vector Machines (SVM), Gradient Boosting Trees (GBT), and Multi-Layer Perceptron (MLP) models achieving acceptable or superior performance. Figure 6 depicts the overall research process.

### Comparison with the current models

Post-ERCP pancreatitis (PEP) represents one of the most frequent and severe complications associated with ERCP (16). This complication can substantially diminish patients' quality of life, augment healthcare expenditures, and in its gravest manifestations, result in patient mortality (17). Although a multitude of predictive models have been developed that demonstrate adept performance, their implementation is often impeded by their complexity, which hinders their integration into routine clinical practice. Furthermore, there may be an insufficient level of recognition among younger endoscopists regarding the potential risk of PEP occurrence. Therefore, it is necessary to develop a prediction model with acceptable accuracy and relatively straightforward predictors for clinical use. In 2021, Koichi Fujita et al. created a practical scoring system for PEP based on a multicenter study in Japan. Considering the weight of seven predictive factors, they developed a model with an acceptable fit and accuracy (AUROC = 0.791) (18). In 2022, Chan Hyuk Park et al. proposed a model based on high-risk factors before and after ERCP but failed to validate the effectiveness of their preoperative risk prediction model (19). Recently, our group used a logistic regression-based backward algorithm to select predictive factors for the establishment of traditional and machine learning models for PEP. Our machine learning model showed better performance and prospects for clinical application than previous models. Traditional multivariate regression is susceptible to small sample bias, particularly in the context of low probability events such as PEP. This approach may also exhibit limited generalization capabilities when addressing complex nonlinear relationships and instances where positive event samples are scarce. To circumvent these constraints, employing distinct ML algorithm-based models using preoperative and postoperative data can potentially enhance predictive performance and surpass the limitations inherent in existing models (20).

### ML model performance

ML models have been widely applied in the medical field due to their advantages in handling complex nonlinear relationships and high-dimensional data (21). Substantial literatures have discussed the establishment of ML models related to acute pancreatitis. Our group also paid attention to the work published by Livia Archibugi and colleagues in May 2023, which may be the first discussion on using ML to predict the risk of PEP. They innovatively used the SHapley Additive exPlanations (SHAP) method to open the “black box” of algorithms and study how each feature contributes to the model. Unfortunately, neither traditional nor ML models showed AUROC curve values that could be referenced and applied clinically (Gradient Boosting = 0.67, LR = 0.56). They suggested that this might be related to insufficient model training due to data imbalance caused by the low incidence rate (6%) of PEP (22). After augmenting the training set data using the ADASYN method, all models satisfactorily predicted the occurrence of postoperative pancreatitis in the validation set, with XGBoost showing the best AUC: 0.840 (95% Cl 0.7955–0.8851). Meanwhile, the above results also need to be further validated by statistical comparison such as DeLong test using bigger sample size in future. We recognize that ADASYN may induce the risk of overfitting, leading to forecast deviation. Meanwhile, it can indeed effectively improve the performance of classifiers and neural networks when the dataset is highly imbalanced; therefore, it is feasible to use these data to predict PEP events. That is, endoscopists can achieve dynamic prediction based on these features and decide on perioperative strategies, allowing nursing decisions to be more precise and personalized.

### Features for PEP prediction

After ERCP, the interpretation and communication of ML models pose a challenge due to the existence of algorithmic “black boxes”. In this investigation, a model-agnostic interpretive approach was employed to discern the underlying clinical elements that may contribute to the onset of post-endoscopic pancreatitis in patients. Using a sequence backward stepwise regression algorithm, we are instrumental in predicting post we identified key clinical variables that are instrumental in predicting PEP. These variables include the presence of periampullary diverticulum, placement of a pancreatic duct stent, the frequency of guidewire cannulation into the pancreatic duct, non-dilated extrahepatic bile ducts, patient age, and the presence of coronary heart disease. Notably, pancreatic duct stent placement, guidewire passages, non-dilated extrahepatic bile ducts, and patient age are all recognized as either risk or protective factors according to the guidelines set forth by the European Society of Gastrointestinal Endoscopy (ESGE) or the American Society for Gastrointestinal Endoscopy (ASGE) (7, 10, 23). NGP has been used as an independent risk factor for PEP in one recent study (24). In another study, the non-dilated extrahepatic bile ducts factor was found to be a significant predictor for PEP using the comprehensive systematic review and meta-analysis method (25). Periampullary diverticulum may be somewhat controversial. From the data collected, most of the patients with periampullary diverticulum (PAD) included in our study had intra-diverticular papilla or a papilla less than 2 cm from the diverticulum. These diverticula could potentially affect the sphincter of Oddi, which normally serves as the main valve for the pancreaticobiliary tract. In patients with PAD, the presence of a diverticulum often causes the papilla to lose its normal morphology partially or completely, leading to variations in the direction of the pancreaticobiliary ducts (26–28). Additionally, the lack of duodenal smooth muscle support at the site of the diverticulum can lead to reduced tension in the distal walls of the pancreaticobiliary ducts, diminished emptying capacity, and post-sphincterotomy tissue edema exacerbates this condition (29). A compelling piece of evidence is that sphincter of Oddi dysfunction (SOD) has been identified as an important and independent risk factor for PEP in most studies (10). However, regarding coronary heart disease (CHD), current guidelines do not indicate it as an independent protective or risk factor for PEP. Patients with CHD are often on long-term medications, including aspirin. Yet, in our data, the group with CHD exhibited a lower incidence of PEP, aligning with the findings of a study published by Harsh K. Patel et al. They compared 1,374,773 ERCP procedures and found a lower incidence of PEP in patients with a history of myocardial infarction (MI) or coronary revascularization surgery (PCI or CABG) (14.1% vs. 15.4%, *p* < 0.001), though they did not discuss the reasons for this finding in their conclusion (30). In many cases, patients undergoing urgent ERCP for conditions such as common bile duct stones or suppurative cholangitis may not switch to heparin bridging therapy in a timely manner. It is speculated that the lower incidence rate of PEP observed in this patient group could be attributed to the protective effect of nonsteroidal anti-inflammatory drugs (NSAIDs) administered preoperatively to those with CHD against PEP. This reflects the interaction between model variables, and since the Backward selection algorithm is generally more flexible in variable selection, considering more interactions and nonlinear relationships, it is better equipped to capture the complex influences and obtain solid association between CHD and other variables in future (31). It also reminds us that when assessing a patient's PEP risk, it may be necessary to consider the impact of medications routinely taken by the patient on PEP, which could affect the choice of preventive measures and treatment strategies (32).

### Clinical implication and future application

Amidst the extensive discourse on the application and dosage of NSAIDs and other potential prophylactic medications for PEP, as proposed by various clinical guidelines—including those from ESGE, ASGE, and the Chinese Guideline for ERCP (2018 Edition)—it is imperative to consider the evidence underpinning these recommendations. it is questionable how many patients undergo a comprehensive PEP risk assessment preoperatively (2, 10, 33, 34). Therefore, the development of a streamlined predictive model for this population is worthy considering for endoscopists. Initially, a thorough evaluation based on the patient's physical condition and radiological findings should be conducted by endoscopists preoperatively. Patients with pre-existing CHD often took aspirin, which, although found to reduce the incidence of PEP in our data, increases the risk of bleeding due to the continued use of anticoagulants when the sphincterotomy is performed during the ERCP procedure (3, 19). Furthermore, if a periampullary diverticulum is discovered during the procedure, particularly Li-Tanaka type I or II diverticula that severely affect the papillary morphology (35), the relationship between the opening of the papilla and the axis of the bile duct should be carefully considered to choose an appropriate cannulation method to reduce the incidence of postoperative complications (36). In this study, we have identified some unique factors for early identification of PEP, which can provide guidance for future multicenter clinical trials. In future multicenter experiments, with independent validation and more case data, a scoring system for PEP can be established. Based on the confirmed model, we could develop an online risk assessment tool in the future to estimate the risk of PEP in ERCP patients (37). In summary, if patients present with the high-risk factors mentioned above, clinicians should take corresponding measures to improve patient outcomes and expedite discharge.

### Strengths and limitations

This investigation exhibited several notable strengths that warrant emphasis. Firstly, the study implemented a rigorous modeling procedure that encompassed stringent criteria for inclusion and exclusion, exhaustive data processing, meticulous feature selection, model construction, and assessment. This methodological approach yielded a predictive model characterized by streamlined factors yet relatively practical outcomes. Meanwhile, a checklist of reporting guidelines such as transparent reporting of a multivariable prediction model for individual prognosis or diagnosis (TRIPOD) should be performed to improve reproducibility (38). Secondly, this study applied ML methods for the first time to validate the filtered model features, offering higher predictive accuracy than traditional linear models even when dealing with complex data patterns and associations. However, there are several limitations to acknowledge. Primarily, this study utilized a retrospective single-center cohort study due to the limitation of time and resource, limiting the generalizability of this model to other regions due to the differences in patient demographics or procedural practices. Despite the strictest inclusion and exclusion criteria, the small sample size necessitates prospective observational studies for more rigorous validation. The smaller sample size also poses the risk of overfitting in ML models. Secondly, due to the low incidence of PEP postoperatively at our institution, there is a significant imbalance between the samples of PEP patients and non-PEP patients. For model evaluation, precision, recall, and other metrics of the predicted model at different thresholds should be reported besides the AUC assessment. This study relies on stepwise logistic regression for feature screening, which may miss prediction patterns that can only be recognized by nonlinear models (such as high-order interaction terms or threshold effects). Although the final XGBoost model can still partially compensate for such omissions through tree structure, future research should explore ML methods based on regularization to better adapt to the needs of complex models. The interpretability tools for ML models such as SHAP or LIME also should be considered to confirm the model's clinical decision-making basis. Meanwhile, the information about drugs usage was not fully considered in this study because of the limitation of clinical information of patients. Although an adaptive synthetic sampling algorithm is used to balance the sample sizes, external validation is still required in future research. In summary, these limitations call for a large-scale and multicenter cohort study in future to validate this model.

## Conclusion

In conclusion, we developed and validated a streamlined predictive model for PEP, enhancing our understanding of PEP risk factors in our population. The XGBoost and MLP models outperformed other algorithms, highlighting key preoperative and intraoperative variables. These findings can be used to construct specific clinical application scenarios or tool to guide endoscopists in optimizing clinical outcomes for patients with biliary and pancreatic conditions.

## Data Availability

The original contributions presented in the study are included in the article/Supplementary Material, further inquiries can be directed to the corresponding authors.
